# Chorioamnionitis: Case definition & guidelines for data collection, analysis, and presentation of immunization safety data

**DOI:** 10.1016/j.vaccine.2019.05.030

**Published:** 2019-12-10

**Authors:** Alisa Kachikis, Linda O. Eckert, Christie Walker, Azucena Bardají, Frederick Varricchio, Heather S. Lipkind, Khady Diouf, Wan-Ting Huang, Ronald Mataya, Mustapha Bittaye, Clare Cutland, Nansi S. Boghossian, Tamala Mallett Moore, Rebecca McCall, Jay King, Shuchita Mundle, Flor M. Munoz, Caroline Rouse, Michael Gravett, Lakshmi Katikaneni, Kevin Ault, Nicola P. Klein, Drucilla J. Roberts, Sonali Kochhar, Nancy Chescheir, The Brighton Collaboration Chorioamnionitis Working Group

**Affiliations:** a University of Washington, Seattle, WA, USA; b ISGlobal, Hospital Clínic - Universitat de Barcelona, Barcelona, Spain; c Independent Consultant Vaccinologist, Wakefield, RI, USA; d Yale University School of Medicine, New Haven, CT, USA; e Brigham and Women’s Hospital, Boston, MA, USA; f Taiwan Centers for Disease Control, Taipei, Taiwan; g Loma Linda University, Loma Linda, CA, USA; h University of Malawi College of Medicine, Malawi; i Edward Francis Small Teaching Hospital, Banjul, The Gambia; j Medical Research Council - The Gambia at London School of Hygiene and Tropical Medicine, Fajara, The Gambia; k University of The Gambia School of Medicine & Allied Health Sciences, The Gambia; l Medical Research Council: Respiratory and Meningeal Pathogens Research Unit, Johannesburg, South Africa; m Department of Science and Technology National Research Foundation, Vaccine Preventable Diseases, Faculty of Health Sciences, University of the Witwatersrand, Johannesburg, South Africa; n Department of Epidemiology and Biostatistics, Arnold School of Public Health, University of South Carolina, Columbia, SC, USA; o Sanofi Pasteur, USA; p University of North Carolina, Chapel Hill, NC, USA; q Government Medical College, Nagpur, India; r Baylor College of Medicine, Houston, TX, USA; s Indiana University School of Medicine, Indianapolis, IN, USA; t Medical University of South Carolina, Charleston, SC, USA; u University of Kansas Medical Center, Kansas City, KS, USA; v Kaiser Permanente Vaccine Study Centre, Oakland, CA, USA; w Massachusetts General Hospital, Boston, MA, USA; x Global Healthcare Consulting, India; y Erasmus University Medical Center, Rotterdam, the Netherlands

**Keywords:** Chorioamnionitis, Intra-amniotic infection, Amnionitis, Adverse event, Immunization, Guidelines, Case definition

## 1 Preamble

### 1.1 Need for developing case definitions and guidelines for data collection, analysis, and presentation for chorioamnionitis as an adverse event following immunization

**Fig. 1:**
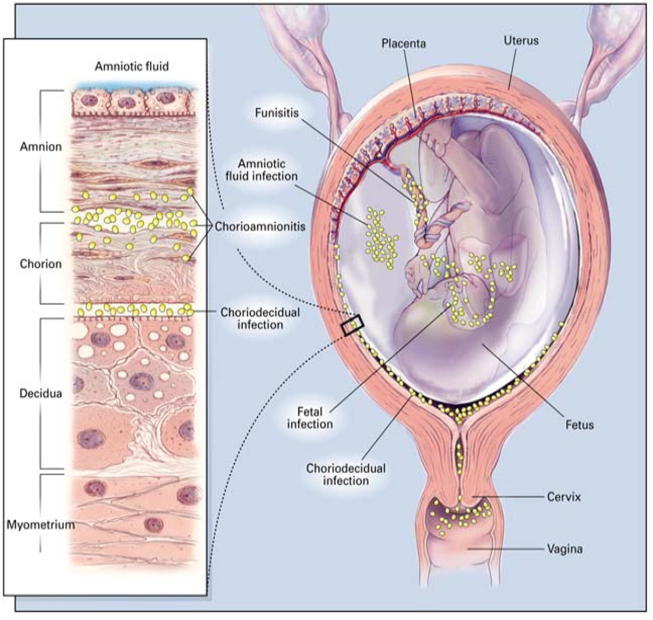
Placental anatomy in the context of intra-amniotic infections (See Section 1.3.4c). Reprinted with permission from Goldenberg RL, Hauth JC, Andrews WW. Intrauterine infection and preterm delivery. N Engl J Med. 2000 May 18;342(20):1500–7 [65].

Chorioamnionitis is a term encompassing a broad spectrum of disease during pregnancy that is characterized by inflammation and/or infection of intrauterine structures such as the placenta, the chorion and amnion (see Fig. 1) [1], [2]. The clinical presentation of chorioamnionitis can vary based on clinical, microbiologic, and histologic factors which interact and overlap to varying degrees [2], [3]. Signs and symptoms depend on whether a primary inflammatory versus an infectious process is found. Placental inflammation is often clinically silent and can signal the normal physiologic process of parturition, an inflammatory process, but can also be a sign of sub-clinical infection. The identification of an infectious etiology, such as a positive amniotic fluid culture, or the development of clinical findings, are indicative of a pathologic process that may progress to more severe maternal and neonatal disease. Distinction of inflammatory versus infectious etiology within the spectrum of chorioamnionitis is therefore important, given the profound differences on subsequent maternal and neonatal morbidity. For the purposes of this case definition we will focus on the infectious manifestation of chorioamnionitis, intra-amniotic infection, and will use these terms interchangeably throughout the remainder of this document. Although chorioamnionitis may affect neonatal morbidity, we will focus on manifestations and pathology found during pregnancy.

*Epidemiology, pathogenesis and risk factors*

Chorioamnionitis or intra-amniotic infection complicates around 1–5% of deliveries at term [4], [5]; however, estimates can vary based on diagnostic criteria used and risk factors [2]. For example, chorioamnionitis can complicate up to one third of pregnancies with preterm labor [6]. The pathogenesis of intra-amniotic infections is most commonly due to ascending infections into the placenta and chorion-amnion [4], [7]. Intrauterine infection can also be transmitted via hematogenous spread as in the case of *Listeria monocytogenes*, or as an iatrogenic infection via procedures for prenatal diagnosis or fetal therapy [4]. There are multiple studies reporting risk factors for chorioamnionitis, which include prelabor rupture of membranes, prolonged labor, nulliparity, internal intrapartum fetal monitoring, multiple vaginal exams, alcohol and tobacco use, bacterial vaginosis, colonization with group B streptococcus, and colonization with *Ureaplasma urealyticum (genital mycoplasmas)* and other pathogens [5], [8], [9], [10], [11], [12].

*Maternal and neonatal outcomes*

Clinically, intra-amnionitic infections can cause significant maternal, fetal and neonatal morbidity and mortality. Women with chorioamnionitis are at higher risk for cesarean section, need for blood transfusion, uterine atony, pelvic abscesses, postpartum endometritis and intensive care unit (ICU) admissions [13], [14], [15]. Severe maternal sequelae of chorioamnionitis can include puerperal sepsis which is an important cause of global maternal mortality both in low- and high-resource settings [16], [17]. Fetal and immediate neonatal consequences of chorioamnionitis include neonatal depression at birth, neonatal sepsis, need for mechanical ventilation, intraventricular hemorrhage, fetal inflammatory response syndrome (FIRS), and neonatal mortality [14], [15], [18]. Preterm neonates are at higher risk for complications than term neonates [15]. With regards to long-term neonatal outcomes, chorioamnionitis is associated with bronchopulmonary dysplasia, periventricular leukomalacia, and cerebral palsy [19], [20].

*Diagnosis of chorioamnionitis*

The diagnosis of chorioamnionitis has been made in previous studies by varying clinical criteria, laboratory, and histologic findings. The presence of inflammation and/or microbes in the placenta, amnion, chorion or amniotic fluid is considered the gold standard for diagnosis, regardless of clinical findings [21], [22], [23], [24]. Unfortunately, histologic examination of the placenta may not be performed if chorioamnionitis is not suspected clinically, and, as such, many studies of chorioamnionitis have not been able to test the specificity of histologic chorioamnionitis. Laboratory tests, such as amniotic fluid culture and glucose analysis, may be limited by exclusion of amniocentesis as a diagnostic test. Even if an amniotic fluid culture is obtained, cultures of certain pathogens such as *Ureaplasma urealyticum* are difficult to perform and not widely available. As well, pathology services and some laboratory assessments, such as microbiologic cultures, are often not readily accessible in all resource settings. These challenges require that we develop a case definition for chorioamnionitis with levels of certainty that are appropriately sensitive and specific for any clinical setting.

Variations in the diagnostic criteria used for chorioamnionitis in the literature make it difficult to interpret individual study results and compare data across studies. Diagnostic criteria for clinical chorioamnionitis are based on early work by Gibbs and colleagues who described chorioamnionitis as maternal fever with two of the following: maternal tachycardia, fetal tachycardia, uterine tenderness, foul odor of amniotic fluid, or maternal leukocytosis [25]. The presence of multiple criteria for clinical chorioamnionitis as well as risk factors has a higher correlation with histologic chorioamnionitis, while individual clinical chorioamnionitis criteria on their own have variable sensitivity and low specificity [2], [23], [26]. Subclinical chorioamnionitis and non-infectious inflammation are within the spectrum of chorioamnionitis described in the literature and likely contribute to discrepancies found between clinical, culture-based and histologic chorioamnionitis (2) [27]. Our case definition does not include these entities.

There are a variety of definitions for chorioamnionitis set forth by international and national health authorities. In their guideline document, the World Health Organization (WHO) defines peripartum infections as “bacterial infection of the genital tract or its surrounding tissues occurring at any time between the onset of rupture of membranes or labor and the 42nd day postpartum in which two or more of the following are present: pelvic pain, fever, abnormal vaginal discharge, abnormal smell/foul odor discharge or delay in uterine involution” [28]. The WHO’s International Classification of Diseases ICD-10 and ICD-11 define chorioamnionitis as O41.12X “Chorioamnionitis” and as JA88.1 “Infection of the amniotic sac and membranes,” respectively [29], [30]. The United Kingdom’s National Institute for Health and Care Excellence (NICE) guidelines for preterm labor does not mention “chorioamnionitis” but does describe prelabor rupture of membranes as risk factor for “intrauterine infection” [31]. The American College of Obstetricians and Gynecologists defines chorioamnionitis as “an infection with resultant inflammation of any combination of the amniotic fluid, placenta, fetus, fetal membranes, or decidua” [32]. While these definitions describe chorioamnionitis, they provide limited guidance regarding diagnostic criteria.

*Vaccines and chorioamnionitis*

With regards to vaccine research, there are several studies that have investigated maternal immunizations and associations with adverse pregnancy outcomes such as chorioamnionitis. A recent systematic review by McMillan et al summarizes antenatal, birth and infant outcomes including chorioamnionitis following maternal immunizations [33]. Four studies are important in the discussion of vaccinations during pregnancy and chorioamnionitis. In 2014, Kharbanda et al published their observational cohort study involving 123,494 women. Chorioamnionitis was diagnosed in 6.1% and 5.5% of women who did and did not receive the tetanus toxoid, diptheria and acellular pertussis (Tdap) vaccine during pregnancy, respectively, demonstrating a small, but significant difference in chorioamnionitis rates (risk ratio [RR] 1.19; 95% confidence interval [CI] 1.13–1.26) [34]. In this study, chorioamnionitis cases were identified retrospectively via ICD-9 codes. Of cases with ICD-9 codes for chorioamnionitis that were randomly selected for chart review, 19.6% of placentas were positive for histologic chorioamnionitis, while 72.3% of placentas did not have a histologic exam. The positive predictive value of a case with ICD-9 code of chorioamnionitis also having “probable” chorioamnionitis (defined as clinical signs of chorioamnionitis with confirmatory histologic chorioamnionitis) was 0.50 (95% CI, 0.43–0.57), and, of note, 95% of women with an ICD-9 code for chorioamnionitis also had an epidural which can be associated with maternal fever.

In 2015, Morgan et al published their retrospective cohort study of 7,378 women and did not find a statistically significant difference in chorioamnionitis rates between those who were and were not vaccinated during pregnancy (odds ratio [OR] 1.51, 95% CI 0.77– 2.96) [33], [35]. The study data was taken from an institutional database of prospectively maintained pregnancy, birth and neonatal records and it is therefore unclear what diagnostic criteria for chorioamnionitis were used in this study [35].

Another retrospective cohort study of 1759 women was published by Berensen et al in 2016. They did not find statistically significant differences in chorioamnionitis rates between those who were and those who were not vaccinated with Tdap during pregnancy (adjusted OR 1.53, 95% CI 0.80–2.90). The diagnosis of chorioamnionitis in this study was based on clinical findings only; neither culture nor histopathologic findings were included for diagnostic purposes [36].

Finally, in 2015, Datwani et al published their review of the Vaccine Adverse Event Reporting System (VAERS) database to explore reports of chorioamnionitis after receipt of any vaccine in the United States between 1990 and 2014. Chorioamnionitis was found to be present in 1% of pregnancy reports to VAERS and most cases had at least one risk factor for chorioamnionitis [37].

The limitations of these studies include that they were all retrospective with risk for various biases. There is also a possibility that these studies were underpowered which decreases the likelihood of finding differences in chorioamnionitis outcomes between women who were vaccinated in pregnancy and women who were not.

Furthermore, based on these studies it is clear that heterogeneity of diagnostic criteria for chorioamnionitis makes it difficult to draw conclusions about whether associations between maternal immunization with Tdap vaccine or any other recommended vaccines during pregnancy and an increased risk of chorioamnionitis actually exist. Each study used varying definitions for chorioamnionitis including ICD-9 codes and clinical symptoms. One study did not specify how chorioamnionitis was defined. This prevents comparison of data across studies and underscores the necessity for a harmonized definition for chorioamnionitis in research studies. Vaccines currently routinely recommended in pregnancy by WHO and national health authorities in the United Kingdom, the United States, Australia, and an increasing number of countries include tetanus toxoid and inactivated influenza vaccines. Most countries recommend acellular pertussis routinely during pregnancy, while WHO recommends the acellular pertussis vaccine specifically in areas of high disease burden [38], [39], [40], [41].

### 1.2 Methods for the development of the case definition and guidelines for data collection, analysis, and presentation for chorioamnionitis as an adverse event following immunization

Due to the lack of a clear definition for chorioamnionitis to facilitate data comparability across trials and surveillance systems, and following the process described in the GAIA overview paper [42] as well as on the Brighton Collaboration Website, https://www.brightoncollaboration.org/about-us/vision-and-mission.html, the Brighton Collaboration Chorioamnionitis Working Group was formed in 2018 and included members from clinical, academic, public health, and industry backgrounds. The composition of the working and reference group can be viewed at: http://www.brightoncollaboration.org.

To guide the decision-making for the case definition and guidelines, a comprehensive literature search was performed by academic library services using Pubmed, Embase and Web of Science. Due to the extensive and diverse topic of chorioamnionitis, the search was limited from 1995 to the date that the search was conducted in February 2018. The search terms for MEDLINE via PubMed is shown below and was modified for Embase and Web of Science search terminology (see Table 1).

**Table 1:** Chorioamnionitis MEDLINE search strategy for the literature search through the academic library.

#1	(“Chorioamnionitis”[Mesh] OR chorioamnionitis[tw] OR amnionitis[tw] OR funisitis[tw])
#2	(Intrauterine infection[tw] OR intrauterine infections[tw] OR intra-uterine infection[tw] OR intra-uterine infections[tw] OR uterine infection[tw] OR uterine infections[tw] OR uterus infection[tw] OR uterus infections[tw] OR intra-amniotic infection[tw] OR intra-amniotic infections[tw]) AND (Pregnancy[mesh] OR pregnancy[tw] OR pregnant[tw])
#3	(“Inflammation”[Mesh] OR inflammation[tw] OR inflammations[tw]) AND (“Extraembryonic Membranes”[Mesh] OR extraembryonic membrane[tw] OR extraembryonic membranes[tw] OR embryo membrane[tw] OR embryo membranes[tw] OR fetal membrane[tw] OR fetal membranes[tw] OR “Amnion”[Mesh] OR amnion[tw] OR amnions[tw] OR amniotic membrane[tw] OR amniotic membranes[tw] OR “Chorion”[Mesh] OR chorion[tw] OR chorions[tw] OR placental membranes[tw] OR placental membrane[tw] OR placenta membrane[tw] OR placenta membranes[tw]) AND (Pregnancy[mesh] OR pregnancy[tw] OR pregnant[tw])
#4	#1 OR #2 OR #3

**Major and Minor Criteria used in the Case Definition of Chorioamnionitis**	
**Major criteria**	
Maternal fever	• At least one measurement of temperature ≥38 degrees Celsius
Pathology	• Findings consistent with histological chorioamnionitis based on a recognized grading system.
Microbiology	• Culture of amniotic fluid or culture of placental membranes between chorion and amnion positive for bacteria
Gestational Age	• ≥22–0/7 weeks estimated gestational age • Prior to complete removal of placenta and membranes either in the third stage of labor or during procedure.
[Absence of]	• Other source of systemic infection (e.g. pyelonephritis, appendicitis, cholecystitis)
**Minor criteria**	
Cardiovascular	● Maternal tachycardia (HR > 100 bpm) ● Fetal tachycardia: Baseline > 160 bpm for 10 min or longer, excluding accelerations, decelerations and periods of marked variability^1^ or, where continuous monitoring is not available, an FHR exceeding 160 bpm over at least three contractions^2^
Genitourinary	● Uterine tenderness ● Purulent fluid from the cervical os or foul smelling amniotic fluid
Laboratory	● Maternal white blood cell count ≥ 15,000 per mm^3^ in the absence of antenatal corticosteroids

1 Correlates with: Ayres-de-Campos D, Spong CY, Chandraharan E, Panel FIFMEC. FIGO consensus guidelines on intrapartum fetal monitoring: Cardiotocography. Int J Gynaecol Obstet. 2015;131(1):13–24.

2 Correlates with: Lewis D, Downe S, Panel FIFMEC. FIGO consensus guidelines on intrapartum fetal monitoring: Intermittent auscultation. Int J Gynaecol Obstet. 2015;131(1):9–12.

The searches in MEDLINE, Embase, and Web of Science yielded a total of 9710 citations. These were exported to Endnote and 2755 duplicates were removed using the Endnote deduplication feature. This left a total of 6955 unique citations found in all searches. The complete strategy for each of the searches can be found in a separate publication. Six committee members (AK, NC, CW, FV, AB, LE) reviewed the search results for appropriateness to the topic via both title/abstract and full text screening, resulting in 194 included documents.

A separate search was done to identify any reports associating chorioamnionitis with vaccinations, using MEDLINE and Embase via the Embase.com platform. Results were limited to English and the preceding 10 year period. The following search string was used:

**Table:** 

(chorioamnionitis'/exp AND 'vaccine'/exp/mj
AND
prenatal tdap immunization and risk of maternal and newborn adverse':ti)
OR
('chorioamnionitis'/exp OR chorioamnionitis:ti,ab) AND ('vaccine'/exp/mj OR vaccin*:ti OR immuniz*:ti OR immunis*:ti)

Another 30 articles related to chorioamnionitis and vaccine were found of which 7 were excluded due to study type. Of the search for chorioamnionitis related to vaccinations, 23 were selected after review.

**Fig. 2:**
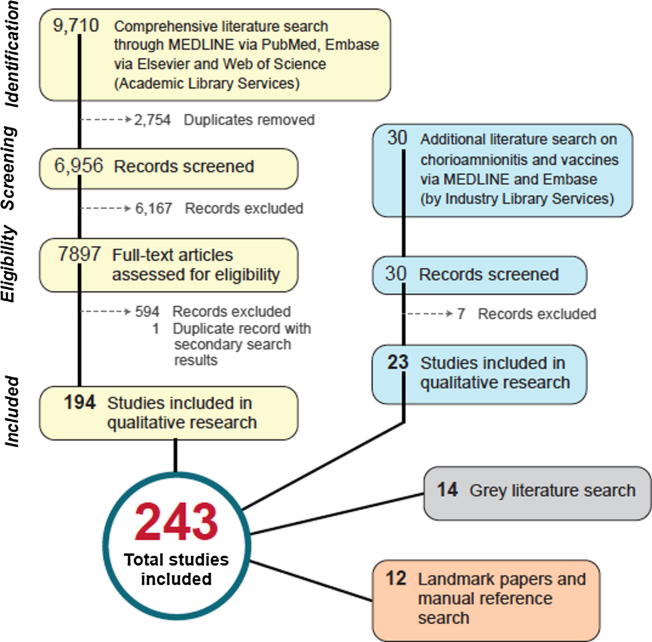
Flow diagram describing pathway for source identification.

In addition, we conducted a grey literature search identifying latest edition text books in common usage in obstetrics and gynecology and national obstetrics and gynecology guidelines: 14 documents were added to the study/documents to review. A manual search of references was conducted for relevant and landmark papers of which 12 studies were selected. A total of 243 studies and documents, including information on a working definition for chorioamnionitis, the appropriate method of diagnosis, or any association of chorioamnionitis as a complication of vaccination, were therefore selected for review. A flow diagram of identified sources is shown in Fig. 2.

### 1.3 Rationale for selected decisions about the case definition of chorioamnionitis as an adverse event following immunization

#### 1.3.1 The term chorioamnionitis

Alternative terminology for chorioamnionitis includes intra-amniotic infection and amnionitis. In the clinical setting with positive clinical signs and symptoms, chorioamnionitis is often referred to as “clinical chorioamnionitis” in the literature. Positive amniotic fluid culture or an elevated amniotic fluid white blood cell count can be referred to as microbial invasion of the amniotic cavity (MIAC) and intraamniotic inflammation, respectively, which are also within the spectrum of chorioamnionitis. Pathologic placental findings consistent with chorioamnionitis are usually referred to as “histologic chorioamnionitis.” More recently the term “triple I” was coined in 2016 by an expert panel workshop at the Eunice Kennedy Shriver National Institute of Child Health and Human Development (NICHD) in the United States to emphasize the full spectrum of the disease as “intrauterine inflammation or infection or both”, however this term is not in wide-spread use within the United States or internationally [1].

#### 1.3.2 Related terms of chorioamnionitis and differentiation of chorioamnionitis from other (similar/associated) disorders

For the purposes of developing this case definition, the focus is on the intraamniotic infectious manifestation of chorioamnionitis that occurs during pregnancy. Related terms of chorioamnionitis that are *not included* in this case definition therefore are:

– Intraamniotic inflammation
– “Triple I”
– Funisitis
– Fetal inflammatory response syndrome
– Septic abortion
– Postpartum endometritis

Intraamniotic inflammation:

• Findings of acute histologic chorioamnionitis with placental invasion of polymorphonuclear cells but without evidence of intraamniotic infection (i.e. negative culture or negative clinical chorioamnionitis) [26].

“Triple I”

• Term coined by the NICHD workshop expert panel to better describe “intrauterine inflammation or infection or both” [1]. This term is not used within this case definition as it can cause confusion, especially outside of the United States, and because the goal of this case definition is to focus on chorioamnionitis as an intra-amnionitic infection.

Funisitis

• Presence of polymorphonuclear cells in fetal structures including the umbilical cord (i.e. the umbilical vessels and/or Wharton’s jelly). Funisitis is the histologic counterpart to Fetal Inflammatory Response Syndrome [27].

Fetal inflammatory response syndrome (FIRS)

• Describes a systemic inflammatory response within the fetus stemming from microbial invasion of the fetus in utero. FIRS correlates with histologic findings of funisitis [27]. It describes the fetal response as opposed to the maternal response as is seen in chorioamnionitis.

Septic abortion (add reference for Rouse C, Gravett M et al)

• Describes evidence of intrauterine infection within 42 days of a competed abortion or a non-viable pregnancy at less than 22 weeks estimated gestational age.^2^

Postpartum endometritis (add reference for Rouse C, Gravett M et al)

• Describes evidence of intrauterine infection within 42 days of a live birth or stillbirth.

#### 1.3.3 Formulating a case definition that reflects diagnostic certainty: weighing specificity versus sensitivity

The focus of this Brighton Collaboration case definition is on chorioamnionitis with three levels of diagnostic certainty. It needs to be emphasized that the grading of definition levels for this case definition is entirely about diagnostic certainty and does not reflect clinical severity of an event. Thus, a clinically severe event may appropriately be classified as Level 2 or 3 rather than Level 1 if it could reasonably be of non-chorioamnionitis etiology. Level 1 diagnostic certainty typically incorporates gold standard diagnostic methods and has the greatest specificity for an adverse event, while Levels 2 and 3 have increasing sensitivity for a disease but decreasing specificity. Detailed information about the severity of the event should always be recorded, as specified by the data collection guidelines. In addition, while a case may not meet the chorioamnionitis case definition diagnostic criteria, the individual woman may still require medical attention and should undergo a thorough medical evaluation or be directed to the nearest health facility.

The number of signs and/or symptoms that will be documented for each case may vary considerably. The case definition has been formulated such that the Level 1 definition is highly specific for the condition. As maximum specificity normally implies a loss of sensitivity, two additional diagnostic levels have been included in the definition, offering a stepwise increase of sensitivity from Level 1 down to Level 3, while retaining an acceptable level of specificity at all levels. In this way it is hoped that all possible cases of chorioamnionitis can be captured.

#### 1.3.4 Rationale for individual criteria or decision made related to the case definition

Based on our literature review, factors important for the diagnosis of chorioamnionitis include clinical, laboratory and microbiology, and pathology findings.

a. Clinical findings

Clinical findings described in published literature that are important for the diagnosis of clinical chorioamnionitis include maternal fever, uterine tenderness, maternal tachycardia, fetal tachycardia, and purulent fluid coming from the cervical os.

Persistent maternal temperature ≥38 degrees Celsius or 100.4 degrees Fahrenheit is considered an abnormal finding during the antepartum and intrapartum period. Elevated maternal temperature can be caused by infectious processes such as chorioamnionitis but has also been found to be associated with non-infectious etiologies including epidural anesthesia and elevated room temperature [43], [44]. Presence of maternal fever is a necessary criterion for the diagnosis of clinical chorioamnionitis [45]. For the purposes of this case definition, we will use the case definition for fever that was previously developed by the Brighton Collaboration which defines fever as temperature ≥38 degrees Celsius on one occasion [46]. Given potentially confounding antepartum and intrapartum factors, it is considered prudent to confirm maternal fever after one finding of elevated temperature.

Chorioamnionitis is also highly associated with maternal tachycardia with heartrate (HR) greater than 100 beats per minute and fetal tachycardia with fetal heartrate (FHR) greater than 160 beats per minute [2]. One study found that 20–80% of chorioamnionitis cases had maternal tachycardia, while 40–70% had fetal tachycardia [45]. There are several non-infectious causes for maternal tachycardia such as medication side effects, hemodynamic changes, and pain; while non-infectious causes for sustained fetal tachycardia are less common, but can include maternal illness, hypoexemia, tachyarrhythmia or prematurity.

Other, more subjective, criteria for chorioamnionitis include uterine tenderness and purulent fluid coming from the cervical os. Uterine tenderness is assessed via physical examination and may be confounded by contraction pain or masked by epidural anesthesia. Purulent fluid coming from the os depends on a speculum examination. Uterine tenderness and purulent fluid from the cervical os were found in 4% to 25% and 5% to 22% of chorioamnionitis cases, respectively [45].

Diagnosis of clinical chorioamnionitis is largely based on two different algorithms. The Gibbs criteria for clinical chorioamnionitis or intraamniotic infection includes maternal fever plus two or more findings of maternal tachycardia, fetal tachycardia, uterine tenderness, foul odor of the amniotic fluid, or maternal leukocytosis [25]. Subsequent studies have, for the most part, used variations of these clinical criteria. A second algorithm for clinical chorioamnionitis was developed during an expert panel workshop at the NICHD in the United States. In this workshop summary, suspected intraamniotic infection (labeled “Triple I”) was defined as maternal fever without a clear source plus one of the following: baseline fetal tachycardia, maternal leukocytosis in the absence of corticosteroids or definite purulent fluid from the cervical os. Confirmed intraamniotic infection (or “Triple I”) was diagnosed with amniocentesis-proven positive gram stain, low amniotic fluid glucose or positive amniotic fluid culture or with placental pathologic features consistent with infection [1].

b. Laboratory findings

Maternal leukocytosis is the laboratory finding most commonly used in the diagnosis of clinical chorioamnionitis. A white blood cell (WBC) count of greater or equal to 15,000/mm^3^ has been used as the cut-off for this criterion [1], [25]. It must be considered that maternal leukocytosis is relatively non-specific and can be induced by several factors including antenatal corticosteroids [2]. Antenatal corticosteroids are especially pertinent since they are often given to patients who are also at high risk for developing chorioamnionitis, such as those with preterm labor and preterm premature rupture of membranes. Other laboratory tests such as C-reactive protein, interleukin-6, soluble intracellular adhesion molecule (sICAM), procalcitonin, lipopolysaccharide binding protein (LBP) and metalloproteinase-8 exist, however, they are of limited value clinically and often used only in research settings [47], [48], [49].

c. Histological findings

The association between histologic findings of chorioamnionitis in the placenta and infection is well established. Positive histologic findings have been found to be more sensitive than clinical chorioamnionitis confirmed via amniotic fluid culture [23], [50]. In addition, histologic chorioamnionitis in term, low-risk pregnancies is often associated with placental inflammation rather than placental infection [26]. The diagnosis of histologic chorioamnionitis is performed retrospectively following childbirth. The diagnostic criteria are based on the stage and grade of maternal polymorphonuclear leukocyte invasion per high-power field into the placental plate and into the membranes, from the chorion to the amnion in an amniotropic direction [51] (see placental anatomy Fig. 1). There are various staging and grading criteria that have been used in the literature regarding pathologic findings of chorioamnionitis within the placenta and membranes and include Redline, Salafia, and Blanc criteria [24], [52], [53]. Redline criteria for diagnosis of histologic chorioamnionitis are outlined in Appendix B.

d. Microbiological findings

While numerous studies have shown correlation between positive amniotic fluid culture and chorioamnionitis, positive fluid cultures can also be found in subclinical infections [21]. Similarly, positive culture results for pathogenic bacteria from swabs between the layers of the placental membrane, the chorion and amnion, correlate with intraamniotic infection [22]. Most intra-amniotic infections are ascending in origin from the genital tract and are polymicrobial, with both anaerobic and aerobic organisms isolated. In one study, women with acute intra-amniotic infection were found to have higher rates of high-virulence isolates compared to controls. These included group B streptococci, *α-*hemolytic streptococci, *Escherichia coli*, *Clostridium spp*, *Bacteroides spp*, among others [25]. Other routes of infection described include hematogenous spread of bacteria such as *Listeria monocytogenes,* group A streptococci and *Campylobacter spp*. The risk of iatrogenic intra-amniotic infection from invasive fetal therapy or prenatal diagnostic procedures is low if appropriate precautions are taken [4]. Other tests on amniotic fluid, including glucose level, lactate dehydrogenase activity, white blood cell count, and gram stain, are less reliable in identifying and confirming chorioamnionitis [1], [54]. Nucleic amplification tests such as polymerase chain reaction (PCR) for detection of intraamniotic infection is used mainly for research purposes [55].

Influence of treatment on fulfilment of case definition

The Working Group decided against using “antibiotic treatment” or “antibiotic treatment response” towards fulfillment of the chorioamnionitis case definition.

A treatment response or its failure is not in itself diagnostic, and may depend on variables such as clinical status, comorbidities, antenatal and intrapartum factors, and other clinical parameters. This is especially pertinent in chorioamnionitis cases since while antibiotics are typically administered to treat clinical signs and symptoms, the ultimate treatment is evacuation of the uterus via childbirth or termination of pregnancy.

#### 1.3.5 Summary of clinical, laboratory, histology and microbiology definitions used for the chorioamnionitis case definition

**Fig. 3:**
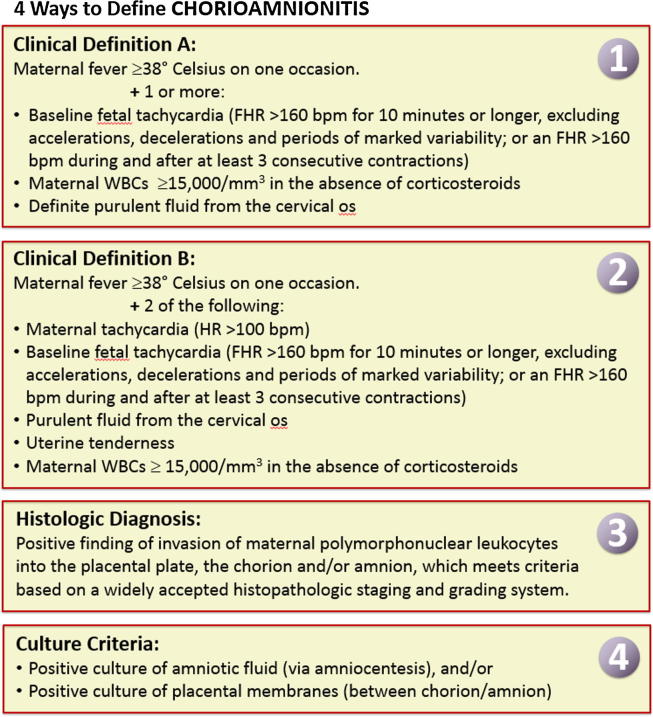
Definitions synthesized for chorioamnionitis based on the systematic literature review. (Two definitions for clinical chorioamnionitis were developed to correlate with the more specific definition for intraamniotic infection (or “Triple I”) from the NICHD chorioamnionitis workship (Clinical definition A), and with the more sensitive Gibbs criteria for chorioamnioitis (Clinical definition B). Please see Section 1.3.4 for further details.)

Based on the discussion in Section 1.3.4, currently available definitions (see Fig. 3) are summarized here for the purpose of developing the chorioamnionitis case definition (See Fig. 3 and Section 2).

It is important to note that Clinical Definition A and Clinical definition B were included as separate entities given their widespread use and historic significance. Neither is considered superior; however, Clinical Definition A is more specific while Clinical Definition B is more sensitive.

**Clinical Definition A**^3^*:

Maternal fever ≥ 38 degrees Celsius on one occasion.^4^

Plus

One or more:

• Baseline fetal tachycardia (FHR > 160 bpm for 10 min or longer, excluding accelerations, decelerations and periods of marked variability^5^ or, where continuous monitoring is not available, an FHR exceeding 160 bpm during and after at least three consecutive contractions^6^)
• Maternal WBC ≥ 15,000 per mm^3^ in the absence of corticosteroids.
• Definite purulent fluid from the cervical os.

**Clinical Definition B**^7^*

Maternal fever ≥ 38 degrees Celsius on one occasion.^8^

Plus

2 of:

• Maternal tachycardia (HR > 100 bpm)
• Baseline fetal tachycardia (FHR > 160 bpm for 10 min or longer, excluding accelerations, decelerations and periods of marked variability^9^ or, where continuous monitoring is not available, an FHR exceeding 160 bpm during and after at least three consecutive contractions^10^)
• Purulent fluid from the cervical os.
• Uterine tenderness.
• Maternal WBC ≥ 15,000 per mm^3^ in the absence of corticosteroids.

**Histologic diagnosis:**

• Positive finding of invasion of maternal polymorphonuclear leukocytes into the placental plate, the chorion and/or amnion which meets criteria based on a widely accepted histopathologic staging and grading system [such as Redline, Salafia, or Blanc criteria [24], [52], [53]].

**Culture criteria:**

• Positive culture of amniotic fluid (via amniocentesis)
• And/or
• Positive culture of placental membranes (between chorion/amnion)

#### 1.3.6 Timing post immunization

Timing considerations diagnosis of chorioamnionitis are important. Chorioamnionitis is largely distinguished from septic abortion based on gestational age of the pregnancy. The Brighton Collaboration definition for abortion is loss of pregnancy at less than or equal to 21-6/7 weeks gestational age [56]. Diagnostic criteria of chorioamnionitis should therefore include a gestational age greater than or equal to 22 completed weeks. In addition, the majority of chorioamnionitis cases occur in the context of preterm labor, prelabor rupture of membranes or prolonged labor at term. This gestational age is based on the Brighton Collaboration spontaneous abortion and ectopic pregnancy guidelines and may not be applicable in all settings [56]. Criteria used for gestational age assessment within this case definition (Section 2) is based on the Brighton Collaboration gestational age assessment algorithm [57]. Chorioamnionitis is diagnosed either prior to childbirth or termination of pregnancy with removal of placenta and membranes or it is diagnosed retrospectively after delivery via pathology examination of the placenta and membranes.

We postulate that a definition designed to be a suitable tool for testing causal relationships requires ascertainment of the outcome (e.g. chorioamnionitis) independent from the exposure (e.g. immunizations). Therefore, to avoid selection bias, a restrictive time interval from immunization to onset of chorioamnionitis should not be an integral part of such a definition. Instead, wherever feasible, details of this interval should be assessed and reported as described in the data collection guidelines.

Further, chorioamnionitis often occurs outside the controlled setting of a clinical trial or hospital. In some settings it may be impossible to obtain a clear timeline of the event, particularly in less developed or rural settings. In order to avoid selecting against such cases, the Brighton Collaboration case definition avoids setting arbitrary time frames.

### 1.4 Guidelines for data collection, analysis and presentation

As mentioned in the overview paper [58], the case definition is accompanied by guidelines which are structured according to the steps of conducting a clinical trial, i.e. data collection, analysis and presentation. Neither case definition nor guidelines are intended to guide or establish criteria for clinical management. Both were developed to improve data comparability.

### 1.5 Periodic review

Similar to all Brighton Collaboration case definitions and guidelines, review of the definition with its guidelines is planned on a regular basis (i.e. every three to five years) or more often if needed.

## 2 Case definition of chorioamnionitis^11^

**Clinical definitions:**

**For all levels of diagnostic certainty**

**Fig. 4:**
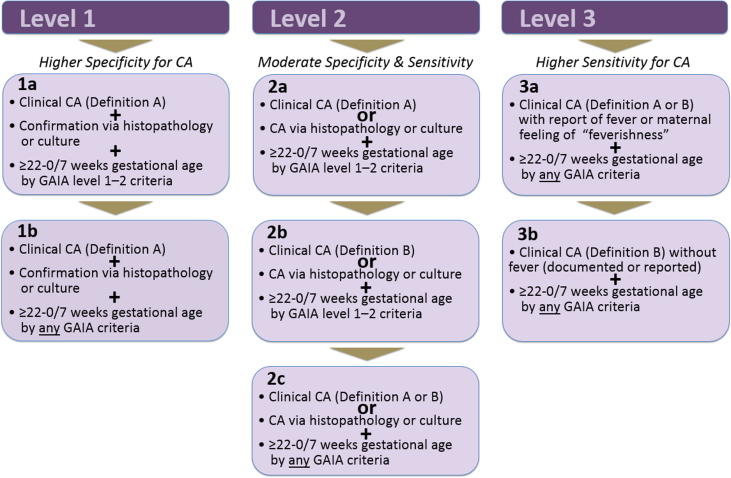
Case definition for chorioamnionitis with levels 1–3 of certainty.
• It is important to rule out other obvious sources of acute systemic infection (i.e. pyelonephritis) prior to chorioamnionitis diagnosis.
• GAIA gestational age level 1–2 criteria denote higher gestational age certainty including a combination of certain last menstrual period (LMP), first or second trimester ultrasound or first trimester exam confirmation. Level 3 diagnostic certainty for gestational age has a lower accuracy compared to levels 1–2. (see Appendix 1)

• *Criteria refer to the factors described in* Section 1.3 *above. Levels of diagnostic certainty for chorioamnionitis in Part 2 should always be interpreted conjointly with the discussion in* Section 1.3.5*. See* Fig. 4*.*

**Level 1 of diagnostic certainty**

**1a7**^12^**:**

Clinical chorioamnionitis (definition A – See Section 1.3.5) with at least one measurement of maternal temperature ≥ 38 degrees Celsius.

**PLUS**

Confirmation via histopathology or culture (See Section 1.3.5)

**PLUS**

Gestational age ≥ 22–0/7 weeks by *GAIA gestational age level 1*–*2 criteria* (See Annex 1)

**1b:**

Clinical chorioamnionitis (definition A – See Section 1.3.5) with at least one measurement of maternal temperature ≥ 38 degrees Celsius.

**PLUS**

Confirmation via histopathology or culture (See Section 1.3.5)

**PLUS**

Gestational age ≥ 22–0/7 weeks *by any GAIA gestational age criteria* (Annex 1)

**Level 2 of diagnostic certainty**

**2a:**

Clinical chorioamnionitis (definition A – See Section 1.3.5) with at least one measurement of maternal temperature ≥ 38 degrees Celsius.

**OR**

Chorioamnionitis via histopathology or culture (See Section 1.3.5)

**PLUS**

Gestational age ≥ 22–0/7 weeks by *GAIA gestational age level 1*–*2 criteria* (Annex 1)

**2b:**

Clinical chorioamnionitis (definition B – see Section 1.3.5) with at least one measurement of maternal temperature ≥ 38 degrees Celsius.

**PLUS**

Gestational age ≥ 22–0/7 weeks by *GAIA gestational age level 1*–*2 criteria* (Annex 1)

**2c:**

Clinical chorioamnionitis (definition A or B – See Section 1.3.5) with at least one measurement of maternal temperature ≥ 38 degrees Celsius.

**OR**

Chorioamnionitis via histopathology or culture (See Section 1.3.5)

**PLUS**

Gestational age ≥ 22–0/7 weeks *by any GAIA gestational age criteria* (Annex 1)

**Level 3 of diagnostic certainty**

**3a:**

Clinical chorioamnionitis (definition A or B – See Section 1.3.5) with report of fever or maternal feeling of “feverishness.”

**PLUS**

Gestational age ≥ 22–0/7 weeks by any GAIA gestational age criteria (Annex 1)

**3b:**

Clinical chorioamnionitis (definition B – See Section 1.3.5) without fever (documented or reported)

**PLUS**

Gestational age ≥ 22–0/7 weeks by any GAIA gestational age criteria (Annex 1)

**Major and Minor Criteria used in the Case Definition of Chorioamnionitis**

## 3 Guidelines for data collection, analysis and presentation of chorioamnionitis

It was the consensus of the Brighton Collaboration *Chorioamnionitis Working Group* to recommend the following guidelines to enable meaningful and standardized collection, analysis, and presentation of information about chorioamnionitis. However, implementation of all guidelines might not be possible in all settings. The availability of information may vary depending upon resources, geographical region, and whether the source of information is a prospective clinical trial, a post-marketing surveillance or epidemiological study, or an individual report of chorioamnionitis. Also, as explained in more detail in the overview paper in this volume, these guidelines have been developed by this working group for guidance only and are not to be considered a mandatory requirement for data collection, analysis, or presentation.

### 3.1 Data collection

These guidelines represent a desirable standard for the collection of data on availability following immunization to allow for comparability of data and are recommended as an addition to data collected for the specific study question and setting. The guidelines are not intended to guide the primary reporting of chorioamnionitis to a surveillance system or study monitor. Investigators developing a data collection tool based on these data collection guidelines also need to refer to the criteria in the case definition (see above), which are not repeated in these guidelines.

Guidelines 1–44 below have been developed to address data elements for the collection of adverse event information as specified in general drug safety guidelines by the International Conference on Harmonization (ICH) of Technical Requirements for Registration of Pharmaceuticals for Human Use [59], and the form for reporting of drug adverse events by the Council for International Organizations of Medical Sciences (CIOMS) [60]. These data elements include an identifiable reporter and patient, one or more prior immunizations, and a detailed description of the adverse event, in this case, of chorioamnionitis following immunization. The additional guidelines have been developed as guidance for the collection of additional information to allow for a more comprehensive understanding of chorioamnionitis following immunization.

#### 3.1.1 Source of information/reporter

For all cases and/or all study participants (including the pregnant woman and/or neonate, as appropriate), the following information should be recorded:

(1) Date of report.
(2) Name and contact information of person reporting^13^ and/or diagnosing chorioamnionitis as specified by country-specific data protection law.
(3) Name and contact information of the investigator responsible for the subject, as applicable.
(4) Relation to the patient (e.g., immunizer [clinician, nurse], family member [indicate relationship], other).

#### 3.1.2 Vaccinee/Control

##### 3.1.2.1 Demographics

For all cases and/or all study participants, as appropriate, the following information should be recorded:

(5) Case/study participant identifiers (e.g. first name initial followed by last name initial) or code (or in accordance with country-specific data protection laws).
(6) Date of birth, age, and sex.
(7) For infants: Gestational age and birth weight.

##### 3.1.2.2 Clinical and maternal immunization history

For all cases and/or all study participants, as appropriate, the following information should be recorded:

(8) Past medical and obstetric history, including hospitalizations, underlying diseases/disorders, infections during pregnancy, pre-immunization signs and symptoms including identification of indicators for, or the absence of, a history of allergy to vaccines, vaccine components or medications; food allergy; allergic rhinitis; eczema; asthma.
(9) Any medication history (other than treatment for the event described) prior to, during, and after immunization including prescription and non-prescription medication as well as medication or treatment with long half-life or long-term effect. (e.g. immunoglobulins, blood transfusion and immunosuppressants such as steroids given to accelerate lung maturity).
(10) Immunization history (i.e. previous immunizations and any adverse event following immunization (AEFI)), in particular occurrence of chorioamnionitis after a previous immunization in pregnancy.
(11) Pregnancy history (i.e. history of or recent preterm labor, preterm premature rupture of membranes, need for cervical cerclage placement or other obstetric procedures), in particular any condition that would increase the risk of chorioamnionitis regardless of whether immunization in pregnancy occurs.

#### 3.1.3 Details of the maternal immunization

For all cases and/or all study participants, as appropriate, the following information should be recorded:

(12) Date, time and place (city/region) of immunization(s).
(13) Description of vaccine(s) (name of vaccine, manufacturer, lot number, dose (e.g. 0.25 mL, 0.5 mL, etc) and number of dose if part of a series of immunizations against the same disease).
(14) The anatomical sites (including left or right side) of all immunizations (e.g. vaccine A in proximal left lateral thigh, vaccine B in left deltoid).
(15) Route and method of administration (e.g. intramuscular, intradermal, subcutaneous, and needle-free (including type and size), other injection devices).
(16) Needle length and gauge.

#### 3.1.4 The adverse event

(17) For all cases at any level of diagnostic certainty and for reported events with insufficient evidence, the criteria fulfilled to meet the case definition should be recorded.

Specifically, document:

(18) Clinical description of signs and symptoms of chorioamnionitis, and if there was medical confirmation of the event (i.e. patient seen by physician or skilled birth attendant).
(19) Date/time of onset^14^, first observation^15^ and diagnosis^16^, end of episode^17^ (i.e. time of delivery or termination of pregnancy) and final outcome^18^ (i.e. development of postpartum endometritis or sepsis, need for further procedures such as hysterectomy, or neonatal outcomes).
(20) Concurrent signs, symptoms, and diseases.
(21) Measurement/testing
  • Values and units of routinely measured parameters (e.g. temperature, blood pressure) – in particular those indicating the severity of the event;
  • Method of measurement (e.g. type of thermometer, oral or other route, duration of measurement, etc.);
  • Results of laboratory examinations, surgical and/or pathological findings and diagnoses if present.
(22) Treatment given for chorioamnionitis, especially specify what antibiotics and additional medications were administered and at what dosing.
(23) Outcome (see Footnote 17) at last observation.
(24) Objective clinical evidence supporting classification of the event as “serious”^19^.
(25) Exposures other than the immunization 24 h before and after immunization (e.g. food, environmental, alternative therapies or tonics) considered potentially relevant to the reported event.

#### 3.1.5 Miscellaneous/ general

(26) The duration of surveillance for chorioamnionitis should be predefined based on
  • Biologic characteristics of the vaccine e.g. live attenuated versus inactivated component vaccines;
  • Biologic characteristics of the vaccine-targeted disease;
  • Biologic characteristics of chorioamnionitis including patterns identified in previous trials (e.g. early-phase trials); and
  • Biologic characteristics of the vaccine (e.g. nutrition, underlying disease like immunosuppressive illness).
(27) The duration of follow-up reported during the surveillance period should be predefined likewise. It should aim to continue to resolution of the event (delivery and the postpartum period).
(28) Methods of data collection should be consistent within and between study groups, if applicable.
(29) Follow-up of cases should attempt to verify and complete the information collected as outlined in data collection guidelines 1 to 25.
(30) Investigators of patients with chorioamnionitis should provide guidance to reporters to optimize the quality and completeness of information provided.
(31) Reports of chorioamnionitis should be collected throughout the study period regardless of the time elapsed between maternal immunization and the adverse event. If this is not feasible due to the study design, the study periods during which safety data are being collected should be clearly defined. However, since chorioamnionitis is immediately followed by delivery or termination of pregnancy, study protocols should make every effort to follow patients until delivery/procedure and through the postpartum or postoperative period in order to capture all chorioamnionitis cases and possible infectious disease sequelae.

### 3.2 Data analysis

The following guidelines represent a desirable standard for analysis of data on chorioamnionitis to allow for comparability of data and are recommended as an addition to data analyzed for the specific study question and setting.

(32) Reported events should be classified in one of the following five categories including the three levels of diagnostic certainty. Events that meet the case definition should be classified according to the levels of diagnostic certainty as specified in the case definition. Events that do not meet the case definition should be classified in the additional categories for analysis.

**Event classification in 5 categories**^20^

**Event meets case definition**

(1) Level 1: Criteria as specified in the chorioamnionitis case definition
(2) Level 2: Criteria as specified in the chorioamnionitis case definition
(3) Level 3: Criteria as specified in the chorioamnionitis case definition

**Event does not meet case definition**

***Additional categories for analysis***

(4) Reported chorioamnionitis with insufficient evidence to meet the case definition^21^
(5) Not a case of chorioamnionitis^22^ (33) The interval between maternal immunization and reported chorioamnionitis could be defined as the date/time of immunization during pregnancy to the date/time of onset (See Footnote 13) of the first symptoms and/or signs consistent with the definition. The time-dependent nature of exposure to vaccination within pregnancy, time-dependent increased risk of events as pregnancy progresses and potential bias such as variable opportunity for vaccination in pregnancy must be considered [61]. If few cases are reported, the concrete time course could be analyzed for each. Examples of increments that could be used for data analysis are as follows:

**Subjects with Chorioamnionitis by Interval to Presentation**

**Table:** 

Interval*	**Number**
<72 h after immunization	
72 - <7 days after immunization	
1 week - <4 weeks after immunization	
4 week increments thereafter until delivery or termination of pregnancy with removal of placenta and membranes either by completion of the third stage of labor or by procedure.	
**Total**

(34) The occurrence of a possible chorioamnionitis case could be analyzed as the interval between the date/time of onset (See Footnote 12) of the first symptoms and/or signs consistent with the definition and the end of episode (See Footnote 16) and/or final outcome (see Footnote 17). Whatever start and ending are used, they should be used consistently within and across study groups. In the case of chorioamnionitis the end of episode may include childbirth or termination of pregnancy with removal of placenta and membranes either during the third stage of labor or via procedure. It must be noted that histologic or culture-positive chorioamnionitis is often diagnosed retrospectively after childbirth or termination of pregnancy has already occurred.
(35) If more than one measurement of a particular criterion is taken and recorded, the value corresponding to the greatest magnitude of the adverse experience could be used as the basis for analysis. Analysis may also include other characteristics like qualitative patterns of criteria defining the event.
(36) The distribution of data (as numerator and denominator data) could be analyzed in predefined increments (e.g. measured values, times), where applicable. Increments specified above should be used. When only a small number of cases is presented, the respective values or time course can be presented individually.
(37) Data on chorioamnionitis obtained from subjects receiving a vaccine should be compared with those obtained from an appropriately selected and documented control group(s) to assess background rates in non-exposed populations

### 3.3 Data presentation

These guidelines represent a desirable standard for the presentation and publication of data on chorioamnionitis following immunization to allow for comparability of data and are recommended as an addition to data presented for the specific study question and setting. Additionally, it is recommended to refer to existing general guidelines for the presentation and publication of randomized controlled trials, systematic reviews, and meta-analyses of observational studies in epidemiology (e.g. statements of Consolidated Standards of Reporting Trials (CONSORT), of Improving the quality of reports of meta-analyses of randomized controlled trials (QUORUM), and of Meta-analysis Of Observational Studies in Epidemiology (MOOSE), respectively) [62], [63], [64].

(38) All reported events of chorioamnionitis should be presented according to the categories listed in guideline 32.
(39) Data on possible chorioamnionitis events should be presented in accordance with data collection guidelines 1–25 and data analysis guidelines 32–37.
(40) Terms to describe chorioamnionitis such as “low-grade”, “mild”, “moderate”, “high”, “severe” or “significant” are highly subjective, prone to wide interpretation, and should be avoided, unless clearly defined.
(41) Data should be presented with numerator and denominator (n/N) (and not only in percentages), if available.

Although immunization safety surveillance systems denominator data are usually not readily available, attempts should be made to identify approximate denominators. The source of the denominator data should be reported and calculations of estimates be described (e.g. manufacturer data like total doses distributed, reporting through Ministry of Health, coverage/population-based data, etc.).

(42) The incidence of cases in the study population should be presented and clearly identified as such in the text.
(43) If the distribution of data is skewed, median and range are usually the more appropriate statistical descriptors than a mean. However, the mean and standard deviation should also be provided.
(44) Any publication of data on chorioamnionitis should include a detailed description of the methods used for data collection and analysis as possible. It is essential to specify:
  • The study design;
  • The method, frequency and duration of monitoring for chorioamnionitis;
  • The trial profile, indicating participant flow during a study including drop-outs and withdrawals to indicate the size and nature of the respective groups under investigation;
  • The type of surveillance (e.g. passive or active surveillance);
  • The characteristics of the surveillance system (e.g. population served, mode of report solicitation);
  • The search strategy in surveillance databases;
  • Comparison group(s), if used for analysis;
  • The instrument of data collection (e.g. standardized questionnaire, diary card, report form);
  • Whether the day of immunization was considered “day one” or “day zero” in the analysis;
  • Whether the date of onset (see footnote 13) and/or the date of first observation (see footnote 14) and/or the date of diagnosis (see footnote 15) was used for analysis; and
  • Use of this case definition for chorioamnionitis, in the abstract or methods section of a publication^23^.

## Disclaimer

The findings, opinions and assertions contained in this consensus document are those of the individual scientific professional members of the working group. They do not necessarily represent the official positions of each participant’s organization (e.g., government, university, or corporation). Specifically, the findings and conclusions in this paper are those of the authors and do not necessarily represent the views of their respective institutions.

## Declaration of Competing Interest

Nicola Klein reports research support from GlaxoSmithKline, Pfizer, Sanofi Pasteur, Merck & Co and Protein Science (now Sanofi Pasteur). Kevin Ault is on several data and safety committees for maternal immunization and drug treatment trials.

## Footnotes

3 “Clinical definition 1” correlates with confirmed intraamniotic infection (or “Triple I”) per the NICHD chorioamnionitis workshop (see Section 1.3.4). *As noted in 1.3.5, Clinical Definition A and B were included as separate entities given their widespread use and historic significance.

4 This correlates with the Brighton Collaboration case definition for fever. Confirmation of fever, however, is recommended (see Section 1.3.4).

7 “Clinical definition 2” correlates with the Gibbs criteria (see Section 1.3.4). *As noted in 1.3.5, Clinical Definition A and B were included as separate entities given their widespread use and historic significance.

8 This correlates with the Brighton Collaboration case definition for fever. Confirmation of fever, however, is recommended (see Section 1.3.4).

11 An event does not meet the case definition if investigation reveals a negative finding of a necessary criterion (necessary condition) for diagnosis. Such an event should be rejected and classified as “Not a case of chorioamnionitis”

12 The difference between levels 1a and 1b is based on diagnostic certainty of gestational age.

20 To determine the appropriate category, the user should first establish, whether a reported event meets the criteria for the lowest applicable level of diagnostic certainty, e.g. Level three. If the lowest applicable level of diagnostic certainty of the definition is met, and there is evidence that the criteria of the next higher level of diagnostic certainty are met, the event should be classified in the next category. This approach should be continued until the highest level of diagnostic certainty for a given event could be determined. Major criteria can be used to satisfy the requirement of minor criteria. If the lowest level of the case definition is not met, it should be ruled out that any of the higher levels of diagnostic certainty are met and the event should be classified in additional categories four or five.

## Footnotes

2 This gestational age cut-off is based on the Brighton Collaboration spontaneous abortion and ectopic pregnancy guidelines and may not be applicable in all settings (Source: Rouse CE, Eckert LO, Babarinsa I, Fay E, Gupta M, Harrison MS, Kawai AT, Kharbanda EO, Kucuku M, Meller L, Mallett Moore T, Subelj M, Kochhar S, Tavares-Da-Silva F; Global Alignment of Immunization Safety in Pregnancy (GAIA) Abortion Work Group; Brighton Collaboration Abortion Working Group. Spontaneous abortion and ectopic pregnancy: Case definition & guidelines for data collection, analysis, and presentation of maternal immunization safety data. Vaccine. 2017 Dec 4;35(48Pt A):6563–6574).

5 This correlates with: Ayres-de-Campos D, Spong CY, Chandraharan E, Panel FIFMEC. FIGO consensus guidelines on intrapartum fetal monitoring: Cardiotocography. Int J Gynaecol Obstet. 2015;131(1):13–24.

6 This correlates with: Lewis D, Downe S, Panel FIFMEC. FIGO consensus guidelines on intrapartum fetal monitoring: Intermittent auscultation. Int J Gynaecol Obstet. 2015;131(1):9–12.

9 This correlates with: Ayres-de-Campos D, Spong CY, Chandraharan E, Panel FIFMEC. FIGO consensus guidelines on intrapartum fetal monitoring: Cardiotocography. Int J Gynaecol Obstet. 2015;131(1):13–24.

10 This correlates with: Lewis D, Downe S, Panel FIFMEC. FIGO consensus guidelines on intrapartum fetal monitoring: Intermittent auscultation. Int J Gynaecol Obstet. 2015;131(1):9–12.

13 If the reporting center is different from the vaccinating center, appropriate and timely communication of the adverse event should occur.

14 The date and/or time of onset is defined as the time post immunization, when the first sign or symptom indicative for chorioamnionitis occurred. This may only be possible to determine in retrospect, especially since onset signs and symptoms of chorioamnionitis can initially be subtle in nature.

15 The date and/or time of first observation of the first sign or symptom indicative for chorioamnionitis can be used if date/time of onset is not known.

16 The date of diagnosis of an episode is the day post immunization when the event met the case definition at any level.

17 The end of an episode is defined as the time the event no longer meets the case definition at the lowest level of the definition (i.e. at the time of childbirth or termination of pregnancy and removal of placenta and membranes via the third stage of labor or procedure).

18 E.g. recovery to pre-immunization health status, spontaneous resolution, therapeutic intervention, persistence of the event, sequelae, death.

19 An AEFI is defined as serious by international standards if it meets one or more of the following criteria: (1) it results in death, (2) is life-threatening, (3) it requires inpatient hospitalization or results in prolongation of existing hospitalization, (4) results in persistent or significant disability/incapacity, (5) is a congenital anomaly/birth defect, (6) is a medically important event or reaction.

21 If the evidence available for an event is insufficient because information is missing, such an event should be categorized as “Reported chorioamnionitis with insufficient evidence to meet the case definition”.

22 An event does not meet the case definition if investigation reveals a negative finding of a necessary criterion (necessary condition) for diagnosis. Such an event should be rejected and classified as “Not a case of chorioamnionitis”.

23 Use of this document should preferably be referenced by referring to the respective link on the Brighton Collaboration website (http://www.brightoncollaboration.org).
