# Supplementary material for: Chorioamnionitis: Case definition & guidelines for data collection, analysis, and presentation of immunization safety data
Source: Vaccine. 2019 Dec 10;37(52):7610–22. doi: 10.1016/j.vaccine.2019.05.030 (PMC6891229; doi:10.1016/j.vaccine.2019.05.030)
Supplement: Supplementary data 2 [file mmc2.docx]

# APPENDIX A: Tool to aid identification of appropriate level of diagnostic certainty

*Case report form*

*or*

*Diagnostic algorithm*

**Annex 1:**

**GAIA Gestational age criteria**

***Prematurity and Assessment of Gestational Age Criteria:***

Quinn J, Munoz FM, Gonik B, Frau L, Cutland C, Mallett-Moore T, Kissou A, Wittke F, Das M, Nunes T, Pye S, Watson W, Alguacil Ramos A, Cordero JF, Huang W, Kochhar S, Buttery J, The Brighton Collaboration Preterm Birth Working Group (2016) Preterm birth: Case definition &amp; guidelines for data collection, analysis, and presentation of immunisation safety data. Vaccine 34(49): 6047-6056.

***Prematurity and Assessment of Gestational Age:***

***Level 1: (highest level of certainty):***

**1**. **Certain LMP * or Intrauterine insemination (IUI) date or Embryo Transfer (ET) date *with* confirmatory 1^st^ trimester scan (≤ 13 6/7 weeks)**

***OR***

**2.** **1^st^ trimester scan (≤ 13 6/7 weeks)**

***Level 2A:***

**1**. **Certain LMP* *with* 2^nd^ trimester scan (14 0/7 weeks to 27 6/7 weeks)***. If LMP and U/S do not correlate, default to U/S GA assessment.*

***OR***

**2**. **Certain LMP* *with* 1^st^ trimester physical examination**

***Level 2B:***

**Uncertain LMP with 2^nd^ trimester scan (14 0/7 weeks to 27 6/7 weeks)**

***Level 3A:***

1. **Certain LMP *with* 3^rd^ trimester scan- 28 0/7 weeks +**

***OR***

**2**. **Certain LMP *with* confirmatory 2^nd^ trimester FH**

***OR***

**3. Certain LMP with birth weight**

***OR***

**4. Uncertain LMP with 1^st^ trimester physical examination**

***Level 3B:***

1. **Uncertain LMP *with* FH.**

***OR***

**2.** **Uncertain LMP *with* newborn physical assessment.**

***OR***

**3.** **Uncertain LMP *with* Birth weight**
