# Supplementary figures and images for: Chorioamnionitis: Case definition & guidelines for data collection, analysis, and presentation of immunization safety data
Source: Vaccine. 2019 Dec 10;37(52):7610–22. doi: 10.1016/j.vaccine.2019.05.030 (PMC6891229; doi:10.1016/j.vaccine.2019.05.030)

# APPENDIX B: Redline criteria for histologic chorioamnionitis staging and grading (52).


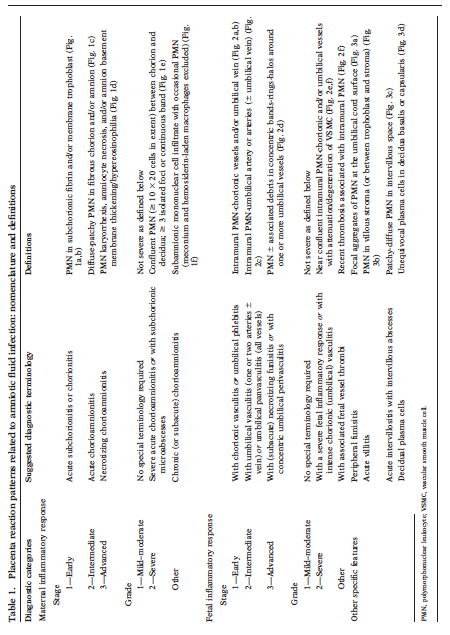

Supplement: Supplementary data 3 [file mmc3.docx]
